# Integrin-Associated Signalling Regulates Jagged1-Induced Osteogenic Differentiation of Human Dental Pulp Stem Cells

**DOI:** 10.1016/j.identj.2026.109808

**Published:** 2026-08-17

**Authors:** Chatvadee Kornsuthisopon, Ajjima Chansaenroj, Suphalak Phothichailert, Betul Gedik, Chengfei Zhang, Nunthawan Nowwarote, Quan Yuan, Lucy Di Silvio, Thanaphum Osathanon

**Affiliations:** a Center of Excellence for Dental Stem Cell Biology, Faculty of Dentistry, Chulalongkorn University, Bangkok, Thailand; b Department of Anatomy, Faculty of Dentistry, Chulalongkorn University, Bangkok, Thailand; c Department of Animal Husbandry, Faculty of Veterinary Science, Chulalongkorn University, Bangkok, Thailand; d Department of Oral and Maxillofacial Surgery, Faculty of Dentistry, Istanbul University, Istanbul, Turkey; e Restorative Dental Sciences, Faculty of Dentistry, The University of Hong Kong, Hong Kong, China; f INSERM UMR1163, Institut Imagine, Université Paris Cité, Paris, France; g State Key Laboratory of Oral Diseases and National Center for Stomatology and National Clinical Research Center for Oral Diseases, West China Hospital of Stomatology, Sichuan University, Chengdu, Sichuan, China; h Center for Oral, Clinical and Translational Sciences, Faculty of Dentistry, Oral & Craniofacial Sciences, King’s College London, London, UK

**Keywords:** Cilengitide, Human dental pulp stem cells, Integrin signalling, Jagged1, Osteogenic differentiation, Notch signalling

## Abstract

**Aim:**

To investigate the role of integrin-associated adhesion signalling in Jagged1-driven osteogenic differentiation and to determine the specific contribution of integrin β3 (ITGβ3).

**Methodology:**

RNA sequencing data from Jagged1-activated human dental pulp cells were analysed to identify changes in integrin-associated signalling. Jagged1-induced Notch activation and ITGβ3 expression were validated by quantitative real-time polymerase chain reaction and immunofluorescence. Functional inhibition of integrin activity was performed using the αvβ3 antagonist cilengitide, and the specific role of ITGβ3 was examined using ITGβ3-targeting siRNA. Apoptosis was evaluated by flow cytometry, migration by a wound-healing assay, and osteogenic differentiation by Alizarin Red S staining and osteogenic gene expression.

**Results:**

RNA sequencing analysis revealed enrichment of extracellular matrix- and integrin-associated pathways among Jagged1-responsive genes in human dental pulp stem cells (hDPSCs), with ITGβ3 identified as a prominently upregulated integrin subunit. Jagged1 induced *ITGB3* mRNA expression in parallel with dose- and time-dependent increases in the Notch target genes *HES1* and *HEY1*, and increased ITGβ3 protein expression was further confirmed by immunofluorescence. Pharmacological inhibition with cilengitide did not affect viability, proliferation, or migration, but significantly suppressed basal and Jagged1-induced mineralisation and reduced *RUNX2* expression. High-dose cilengitide (1000 nM) increased late-stage apoptosis under Jagged1 stimulation. In contrast, *ITGB3* silencing attenuated basal osteogenic differentiation but did not impair Jagged1-induced mineralisation.

**Conclusion:**

Jagged1-induced osteogenic differentiation of hDPSCs requires integrin-dependent adhesion signalling but is not exclusively dependent on *ITGB3* transcription. These findings support integrin–Notch crosstalk as a regulatory mechanism in hDPSC osteogenesis and provide a rationale for designing regenerative dental biomaterials that combine Notch-activating and adhesion-supportive cues.

## Introduction

Osteogenic differentiation of mesenchymal stem cells (MSCs) is regulated by the coordinated activation of multiple signalling pathways, including transforming growth factor-β, bone morphogenetic protein, Wnt/β-catenin, and Notch signalling pathways.^1^ These tightly coordinated molecular networks regulate stem cell fate determination, proliferation, and lineage specification during tissue regeneration and repair processes.^1^, ^2^, ^3^, ^4^, ^5^ Thus, modulation of these pathways can shift the balance between MSC self-renewal and lineage commitment.

Among these regulatory systems, Notch signalling plays an important role in stem cell homeostasis and cell-fate regulation. It also participates in a broad range of biological functions, including apoptosis, tissue turnover, and regenerative responses.^6^, ^7^, ^8^ Activation of the Notch pathway requires direct cell–cell contact between ligand-expressing and receptor-bearing cells.^9^ Ligand binding triggers sequential proteolytic cleavage of the Notch receptor, leading to the release of the Notch intracellular domain. The Notch intracellular domain subsequently translocates into the nucleus, where it regulates transcription of downstream target genes such as Hairy and enhancer of split 1 (*HES1*) and Hairy/enhancer-of-split related with YRPW motif (*HEY1*), which are critical mediators of Notch-dependent cellular responses.^10^^,^^11^

Among the canonical Notch ligands, Jagged1 has emerged as an important modulator of mineralised tissue formation and osteogenic differentiation. Previous studies have consistently demonstrated that Jagged1 markedly enhances odonto/osteogenic differentiation across multiple dental-derived stem cell populations, including human dental pulp stem cells (hDPSCs), stem cells from human exfoliated deciduous teeth, and human periodontal ligament stem cells.^11^, ^12^, ^13^, ^14^ These findings support the relevance of Jagged1-mediated Notch activation to dental stem cell differentiation and mineralisation.

Importantly, the biological activity of Jagged1 is highly dependent on its mode of presentation. Indirect immobilisation of Jagged1 has been shown to induce stronger Notch activation compared with direct ligand coating. This strategy significantly increases C-promoter binding factor-1-dependent transcriptional activity and promotes dose-dependent upregulation of Notch target genes in both epithelial and dental-derived stem cells.^15^^,^^16^ Correspondingly, indirect Jagged1 presentation markedly enhances osteogenic differentiation and mineral deposition in human periodontal ligament stem cells and stem cells from human exfoliated deciduous teeth.^11^^,^^14^^,^^16^ Proteomic profiling of Jagged1-activated hDPSCs further revealed enrichment of odontogenesis-related biological processes under osteogenic conditions, supporting the involvement of Jagged1-driven Notch signalling in mineralised tissue formation.^17^

Integrins constitute a major family of transmembrane adhesion receptors that mediate interactions between cells and the extracellular matrix (ECM), and in some contexts contribute to cell–cell communication. Through these interactions, integrins regulate a wide array of cellular behaviours, including cell survival,^18^ migration,^19^ and lineage commitment.^20^ Structurally, integrins function as heterodimeric complexes composed of α and β subunits, whose specific pairings determine ligand affinity and intracellular signalling potential.^21^^,^^22^ The binding of integrins to ECM components such as fibronectin, collagen, and laminin enables bidirectional signal transmission between the extracellular environment and the cytoskeleton, thereby influencing cellular tension, mechanotransduction, and differentiation.^23^^,^^24^ Several integrin subunits, including α2β1, αvβ3, α5β1, and β3- or β5-containing integrins, have been implicated in osteoblast differentiation and bone remodelling through their ability to transduce biomechanical and biochemical cues from the ECM.^25^, ^26^, ^27^

Despite the established pro-osteogenic role of Jagged1 in dental-derived stem cells, the downstream cellular mechanisms that cooperate with Notch signalling to support Jagged1-induced osteogenic differentiation remain incompletely defined. In particular, the contribution of cell–matrix adhesion and integrin-associated signalling to this process has not been fully investigated. Previous transcriptomic profiling of hDPSCs stimulated with immobilised Jagged1 revealed enrichment of gene clusters related to ECM organisation, cell adhesion and intracellular signalling pathways, suggesting that Jagged1–Notch activation may influence adhesion-associated regulatory networks.^16^ However, that study did not identify which specific adhesion receptors or integrin subtypes participate in mediating Jagged1-driven osteogenic responses.

Among candidate integrin subunits, integrin β3 (ITGβ3) is of particular interest because β3-containing integrins have been implicated in osteogenic differentiation, adhesion-dependent survival and matrix mineralisation.^18^^,^^28^ Integrins also function as mechanotransducers that link ECM cues to intracellular signalling during osteogenic differentiation.^22^^,^^23^ In particular, integrin-associated focal adhesion kinase (FAK)/extracellular signal-regulated kinase (ERK) signalling has been reported to regulate Runt-related transcription factor 2 (RUNX2) expression and osteogenic differentiation.^28^^,^^29^ Based on this rationale, we hypothesised that integrin-associated adhesion signalling may act as a permissive or cooperative regulator of Jagged1-induced osteogenesis in hDPSCs.

Accordingly, the present study was designed to extend previous findings on the pro-osteogenic effect of Jagged1 in dental-derived stem cells by investigating whether integrin-associated adhesion signalling contributes to Jagged1-induced osteogenic differentiation of hDPSCs and by clarifying the specific involvement of ITGβ3. By integrating transcriptomic reanalysis with pharmacological inhibition of integrin activity, targeted ITGβ3 silencing, protein-level validation of ITGβ3 expression and assessment of ERK signalling as an early downstream readout, this study examined whether Jagged1-induced osteogenic differentiation is supported by broader functional integrin-associated signalling or depends specifically on ITGβ3 expression.

## Materials and methods

### Primary culture of hDPSCs

The cell isolation protocol has been approved by the Human Ethical Research Committee, Faculty of Dentistry, Chulalongkorn University (approval No. 002/2025). Impacted third molars scheduled for surgical extraction were obtained from healthy adult donors with informed consent. Donor recruitment was not restricted by sex. hDPSC cultures were established from independent healthy adult donors and expanded separately. Each donor-derived culture was considered an independent biological replicate. Tissue explantation was employed to isolate the cells. Briefly, pulp tissues were gently removed and minced into small pieces, then placed in 35-mm tissue culture dishes containing 1000 µL of growth medium (GM). GM was composed of Dulbecco’s Modified Eagle Medium (cat. no. 11960, Gibco, Thermo Fisher Scientific) supplemented with 10% foetal bovine serum (cat. no. 10270, Gibco), 1% l-glutamine (GlutaMAX-1, cat. no. 35050, Gibco), 100 units/mL penicillin, 100 µg/mL streptomycin, and 250 ng/mL amphotericin B (Antibiotic–Antimycotic, cat. no. 15240, Gibco). Upon reaching confluence, cells were trypsinized and seeded into 60-mm tissue culture dishes, considered passage 1. Subculturing was performed at a 1:3 ratio upon cell confluence. The cells in passages 3 to 6 were employed for subsequent experiments. All cultures were maintained at 37°C in a humidified atmosphere with 5% CO_2_. Cilengitide (cat. no. SML1594, Sigma-Aldrich), a selective αvβ3-integrin antagonist, was added at concentrations ranging from 3 to 1000 nM.

### Stem cell characterisation

Surface marker expression was analysed using flow cytometry. Single-cell suspensions were stained with the following antibodies at a 1:50 dilution: FITC-conjugated antihuman CD44 (cat. no. 555478, BD Biosciences), FITC-conjugated antihuman CD90 (cat. no. ab124527, Abcam), PE-conjugated antihuman CD105 (cat. no. 21271054, ImmunoTools), and PerCP-conjugated antihuman CD45 (cat. no. 21810455, Immuno Tools). Data were acquired using a FACS^Calibur^ flow cytometer and analysed with CellQuest software (BD Biosciences).

Multilineage differentiation towards osteogenic and adipogenic lineages was determined. For osteogenic differentiation, the cells (25,000 cells/well in a 24-well plate) were cultured in an osteogenic medium consisting of GM supplemented with 50 µg/mL ascorbic acid (cat. no. A-4034, Sigma-Aldrich), 250 nM dexamethasone (cat. no. D8893, Sigma-Aldrich), and 5 mM β-glycerophosphate (cat. no. G9422, Sigma-Aldrich) for 14 days. Calcium deposits were detected using specific staining as described below. As for the adipogenic differentiation, the cells (12,500 cells/well in a 24-well plate) were maintained in an adipogenic medium comprising GM containing 0.1 mg/mL insulin (cat. no. 11070738 Sigma-Aldrich), 1 µM dexamethasone (cat. no. D8893, Sigma-Aldrich), 1 mM IBMX (cat. no. PHZ1124, Gibco), and 0.2 mM indomethacin (cat. no. 53861, Sigma-Aldrich) for 16 days. The intracellular lipid accumulation was visualised by Oil Red O staining.

### Indirect Jagged1 immobilisation

Jagged1 was immobilised indirectly as previously reported.^11^ Briefly, 24-well tissue culture plates were incubated with 50 μg/mL recombinant protein G (cat. no. 101201, Invitrogen) for 16 hours, followed by blocking with 10 mg/mL bovine serum albumin solution (cat. no. A9418, Sigma-Aldrich) for 2 hours. Plates were then incubated with 0.1, 1, or 10 nM rhJagged1/Fc recombinant protein (cat. no. 1277-JG, R&D Systems). Control wells were coated with equivalent concentrations of human IgG Fc (hFc) fragment (cat. no. 009000008, Jackson Immuno Research Labs). Plates were washed with sterile phosphate-buffered saline (PBS) between each step. For selected experiments, cells were cultured on 10 nM immobilised Jagged1 for 24 hours with or without pretreatment with the Notch signalling inhibitor DAPT. hFc-coated wells were used as controls. Gene expression of canonical Notch target genes and endogenous Notch pathway components was evaluated by quantitative real-time polymerase chain reaction (qPCR).

### Transfection with *ITGB3* siRNA

Cells were transfected with Silencer Select Pre-Designed *ITGB3* siRNA (cat. no. 4392420; assay ID s7580, Thermo Fisher Scientific) or Silencer Select Negative Control siRNA (cat. no. 4390843, Thermo Fisher Scientific) using Lipofectamine 2000 Transfection Reagent (cat. no. 11668019, Thermo Fisher Scientific) according to the manufacturer’s protocol for 24 and 72 hours.

### Immunofluorescence staining for ITGβ3

Immunofluorescence staining was performed to evaluate ITGβ3 protein expression in hDPSCs following Jagged1 stimulation and to validate *ITGB3* knockdown at the protein level. For Jagged1 stimulation experiments, hDPSCs were cultured on hFc- or immobilised Jagged1-coated surfaces for the indicated time period. For knockdown validation, hDPSCs were transfected with control siRNA or *ITGB3*-targeting siRNA before immunofluorescence staining.

Cells were fixed with 4% paraformaldehyde for 15 minutes at room temperature, washed with PBS, and permeabilised with 0.1% Triton X-100 for 10 minutes. Nonspecific binding was blocked with 1% bovine serum albumin in PBS for 1 hour at room temperature. Cells were then incubated with primary antibody against ITGβ3 (cat. no. ab197662, Abcam) at a 1:100 dilution at 4°C overnight, followed by incubation with Alexa Fluor 594-conjugated secondary antibody (cat. no. ab150080, Abcam) at a 1:2000 dilution for 1 hour at room temperature in the dark. Nuclei were counterstained with DAPI. Images were acquired using a fluorescence microscope under identical acquisition settings within each experiment.

### Western blot assay

To assess downstream adhesion-associated signalling, western blot analysis was performed to evaluate ERK activation. Following the indicated treatments, hDPSCs were lysed in RIPA buffer supplemented with protease inhibitor buffer (cat. No. P8340, Sigma-Aldrich). The concentration of lysate proteins was measured with a BCA protein assay (PierceTM BCA detection reagent, cat No. 23228 and 23224, Thermo Scientific). Lysate proteins were separated on 12% SDS-polyacrylamide gel and transferred to a nitrocellulose membrane. The membrane was placed in a solution containing a monoclonal antibody to rabbit-antiactin (cat. No. A2066, Sigma-Aldrich), rabbit anti-ERK1/2 (T202/Y204, Cell Signaling), or rabbit anti-phosphorylated ERK1/2 (cat. No. 137F5, Cell Signaling) antibodies. Then, the membranes were developed with the horseradish peroxidase-linked antibody (cat No. 7074S, Cell Signaling). The membranes were visualised and exposed to chemiluminescence (SuperSignal West Pico Chemiluminescent Substrate, cat No. 34577, Thermo Scientific) and an image analyser (GE Healthcare), respectively. The band density was measured using ImageJ software. The band density was normalised to band density of actin and to the control.

### Oil red O staining

Cells were fixed with 10% buffered formalin for 30 minutes and stained with 0.2% Oil Red O solution for 15 minutes. Lipid droplets were visualised using an inverted microscope (Olympus America Inc).

### Mineralisation assay

Cells were fixed with cold methanol for 10 minutes and stained with 2% Alizarin Red S (Sigma-Aldrich) for 3 minutes at room temperature. Stained calcium deposits were solubilised with 10% cetylpyridinium chloride in 10 mM sodium phosphate. Absorbance was measured at 570 nm using a microplate reader (Molecular Devices).

### Cell viability and proliferation assay

Cell proliferation was indirectly determined using the colourimetric MTT (3-[4,5-dimethylthiazol-2yl] diphenyltetrazolium bromide) assay. Cells were seeded into 96-well plates at a density of 3000 cells/well and allowed to attach overnight at 37°C in a humidified atmosphere containing 5% CO_2_. Then, cells were stimulated with different concentrations of cilengitide for 1, 3, and 7 days. At each desired time point, cells were treated with 0.5 mg/mL MTT solution (USB Corporation) for 15 minutes at 37°C. Formazan crystals were dissolved in DMSO-glycine buffer, and absorbance was measured at 540 nm (Molecular Devices). The proliferation percentage is directly proportional to the intensity of the purple product.

### Apoptosis assay

Cells (25,000 cells/well in a 24-well plate) were treated with cilengitide for 3 days. Apoptosis was analysed using the FITC Annexin V Apoptosis Detection Kit (cat. no. 640914, BioLegend), followed by flow cytometry analysis (FACS^Calibur^, BD Bioscience).

### Migration assay

Cell migration was performed using an *in vitro* scratch assay. Confluent monolayers cultured in 35-mm dishes were scratched with a 200-μL pipette tip and treated with cilengitide. Images were captured at 0, 24, and 48 hours using an inverted microscope (Olympus). The percentage of cell migration compared with the control was calculated using ImageJ software.

### Quantitative real-time polymerase chain reaction

Total cellular ribonucleic acid (RNA) was isolated using TRIzol reagent (RiboEx solution, cat. no. 301-001, GeneAll, South Korea) and reverse-transcribed using ImProm-II Reverse Transcription System (cat. no. A3800, Promega). qPCR was performed using FastStart Essential DNA Green Master (Roche Diagnostics) on a CFX connect Real-Time PCR machine (Bio-Rad). The amplification profile was as follows: 95°C/20 s, 60°C/20 s, and 72°C/20 s for 45 cycles. Melt curve analysis was performed to determine product specificity. Expression levels were normalised to *GAPDH* and calculated using the 2^−ΔΔ^*^Ct^* method.^30^ The oligonucleotide sequences are shown in Supplementary Table S1.

### Bioinformatic analysis

Raw reads RNA sequencing data of Jagged1-treated hDPSCs were retrieved from the NCBI’s Gene Expression Omnibus database (GSE94989).^16^ The dataset included hFc control, 10 nM immobilised Jagged1-treated cells for 24 hours, and Jagged1-treated cells pretreated with DAPT, with three biological replicates per group. The original dataset was generated using Illumina NextSeq 500 sequencing, and processed gene expression values were reported as FPKM values following read quality control, mapping to the human reference genome, and transcript abundance estimation.

For the present reanalysis, the processed gene expression matrix was analysed using NetworkAnalyst.^31^ Genes with an adjusted *P* value <.05 were considered statistically significant. No additional fold-change cutoff was applied; however, log2 fold-change values were used to indicate the direction and magnitude of gene expression changes. Expression patterns of selected ECM- and integrin-associated genes across hFc, Jagged1 and Jagged1 + DAPT groups were visualised as a heatmap using Heatmapper.^32^ This targeted gene-level reanalysis was used to identify candidate integrin subunits for subsequent qPCR validation and functional experiments.

### Statistical analysis

All experiments were performed using independent donor-derived hDPSC cultures. The number of biological replicates used for each experiment is indicated in the corresponding figure legends. Biological replicates represent cells derived from independent donors. Due to the relatively small sample sizes and the use of primary donor-derived cultures, normal distribution was not assumed. Therefore, nonparametric tests were used for statistical analysis. The Mann–Whitney *U* test was used for two-group comparisons, and the Kruskal–Wallis test with post hoc pairwise comparisons was used for multiple-group comparisons. An exploratory Spearman correlation analysis was performed to assess the relationship between Notch target gene expression and *ITGB3* expression in Jagged1-treated samples collected during the time-course experiment. Statistical significance was considered at *P* < .05.

## Results

### Stem cell characterisation

The cells isolated from dental pulp tissue exhibited a spindle-shaped, fibroblast-like morphology. Flow cytometric analysis confirmed a MSC-like phenotype, as the cells were positive for CD44, CD90 and CD105, and negative for the haematopoietic marker CD45 (Figure S1A). Multilineage differentiation assays further confirmed the stem cell properties of the isolated cells. Following osteogenic induction for 14 days, mineralised nodules were detected by Alizarin Red S staining (Figure S1B). Under adipogenic conditions for 16 days, intracellular lipid droplet accumulation was observed by Oil Red O staining (Figure S1C).

### Jagged1 induced *ITG3* expression in hDPSCs

**Fig. 1:**
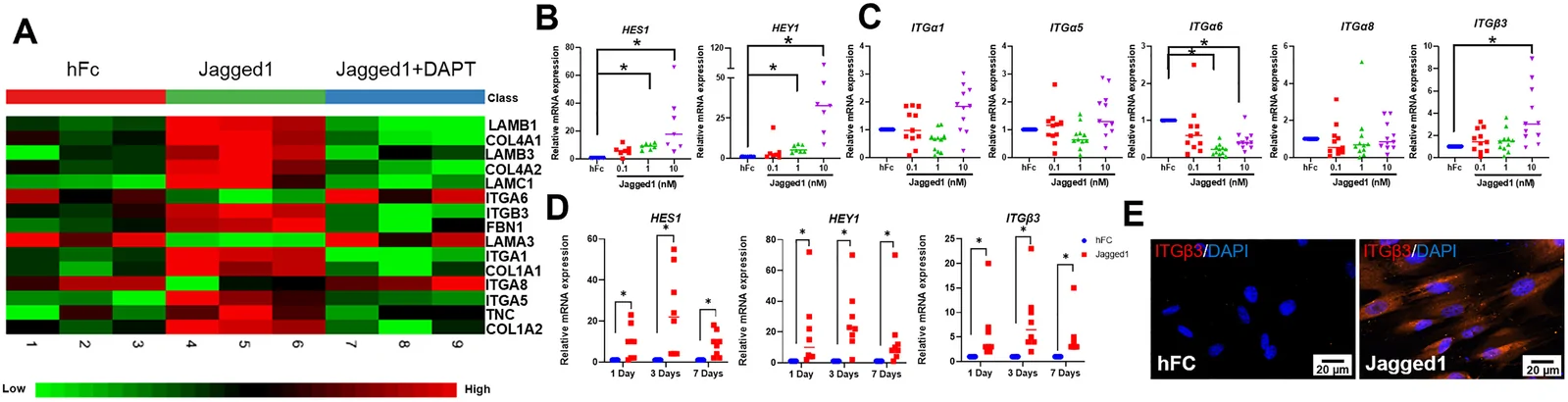
Jagged1 induces integrin-associated gene expression and *ITGB3* expression in hDPSCs. (A) Heatmap illustrating differentially expressed integrin-related genes derived from reanalysis of RNA sequencing data of hDPSCs treated with 10 nM Jagged1 for 24 hours (GSE94989). Integrin-associated pathway modulation was abolished by pretreatment with the Notch inhibitor DAPT, indicating Notch-dependent regulation. (B) qPCR analysis of Notch target and (C) integrin-related genes following treatment with indirect immobilised Jagged1 (0.1, 1, and 10 nM). (D) Time-course analysis (days 1, 3, and 7) of mRNA expression of Notch target genes and *ITGB3.* (E) Representative immunofluorescence images showing enhanced ITGβ3 protein expression in hDPSCs cultured on 10 nM Jagged1-coated surfaces compared with hFc controls (scale bar = 20 μm). Bars indicate a significant difference between the treated groups and the control group (*P < .05). Biological replicates were independent donor-derived hDPSC cultures. The number of biological replicates was as follows: (A) *n* = 3 per group from GSE94989; (B) *n* = 7; (C) *n* = 11; (D) *n* = 8; (E) representative images from *n* = 4.

Transcriptomic analysis was performed by reanalysis of RNA sequencing data previously generated from human dental pulp cells treated with 10 nM Jagged1 for 24 hours, retrieved from the GEO database (GSE94989). The original RNA-sequencing study reported that immobilised Jagged1 stimulation altered gene clusters related to ECM organisation, cell adhesion and intracellular signalling in hDPSCs. Based on this pathway-level observation, we performed a targeted gene-level reanalysis of this dataset to examine ECM- and integrin-associated genes. Heatmap analysis showed that several ECM- and integrin-related genes were altered in Jagged1-treated hDPSCs compared with hFc controls, and these changes were attenuated in the Jagged1 + DAPT group (Figure 1A). Among the integrin subunits examined, *ITGB3* was one of the most prominently upregulated candidates, supporting its selection for subsequent validation and functional analysis. hDPSCs were treated with indirect immobilised Jagged1 at concentrations of 0.1, 1, and 10 nM and maintained in GM for 24 hours. qPCR validation demonstrated that Jagged1 treatment dose-dependently increased the expression of Notch target genes *HES1* and *HEY1*, confirming Notch pathway activation (Figure 1B). Among integrin subunits, *ITGA6* expression was significantly downregulated at 1 and 10 nM Jagged1, while *ITGB3* expression was significantly upregulated at 10 nM Jagged1. In contrast, *ITGA1, ITGA5*, and *ITGA8* expression levels were not significantly altered (Figure 1C).

Time-course analysis further showed sustained upregulation of *HES1* and *HEY1* on days 1, 3, and 7, which corresponded with increased *ITGB3* expression over the same period (Figure 1D). To further examine whether Jagged1-induced *ITGB3* expression was associated with Notch target gene expression during the time-course experiment, an exploratory Spearman correlation analysis was performed using Jagged1-treated samples collected on days 1, 3, and 7. *ITGB3* expression positively correlated with both *HES1* expression (Spearman *r* = 0.86, *P* < .001) and *HEY1* expression (Spearman *r* = 0.91, *P* < .001) (Figure S2A, B). These findings support an association between Notch target gene expression and *ITGB3* expression during the 7-day observation period, although this analysis does not establish a causal relationship. Consistently, immunofluorescence staining confirmed enhanced ITGβ3 protein expression in cells cultured on 10 nM Jagged1-coated surfaces compared with hFc controls (Figure 1E). To further examine endogenous Notch pathway components, *JAG1* and *NOTCH1* expression were evaluated in hDPSCs cultured on 10 nM immobilised Jagged1 for 24 hours, with or without DAPT pretreatment. Endogenous *JAG1* (Figure S3A) and *NOTCH1* (Figure S3B) expression were not significantly altered under these conditions, suggesting that the observed Notch activation was primarily driven by immobilised recombinant Jagged1 rather than transcriptional upregulation of endogenous Notch ligand or receptor expression.

### Integrin inhibition by cilengitide attenuated osteogenic differentiation of hDPSCs

**Fig. 2:**
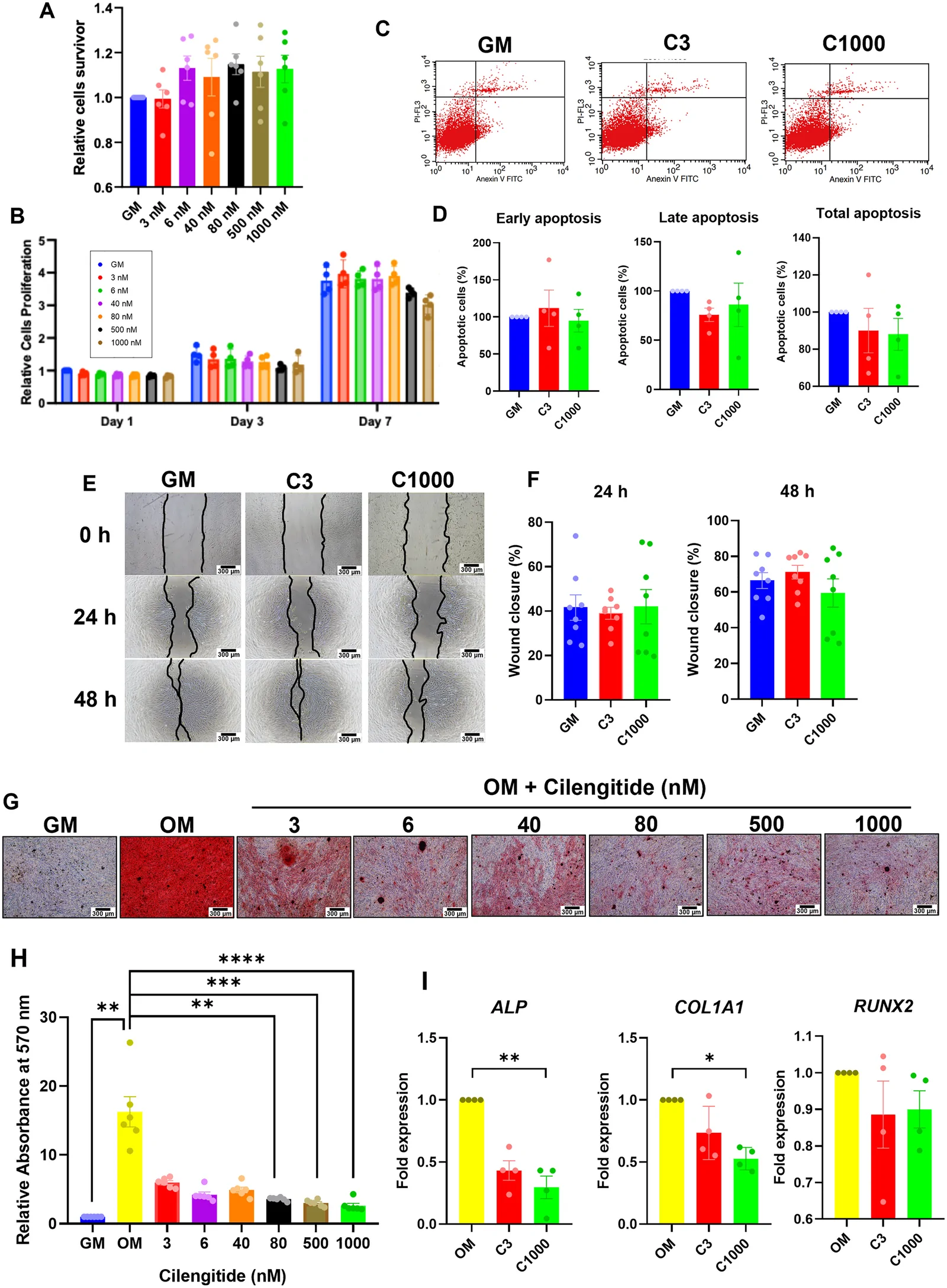
Effect of integrin inhibition by cilengitide on viability, proliferation, apoptosis, migration and osteogenic differentiation of hDPSCs. (A) Cell viability of hDPSCs treated with increasing concentrations of cilengitide (3-1000 nM), as assessed by MTT assay. (B) Cell proliferation of hDPSCs following cilengitide treatment at days 1, 3, and 7. (C and D) Apoptotic activity of hDPSCs treated with cilengitide under basal culture conditions on day 3, as determined by flow cytometry. (E and F) Cell migration of hDPSCs was evaluated using the scratch wound assay at 24 and 48 hours after cilengitide treatment. (G) Alizarin Red S staining showing mineral deposition after 14 days of osteogenic induction with cilengitide treatment. (H) Quantitative analysis of mineralisation following cilengitide treatment. (I) qPCR analysis of osteogenic-related gene expression on day 7 following cilengitide treatment. Bars indicate significant differences between the treated groups and the control group (*P < .05, **P < .01, ***P < .001). Biological replicates were independent donor-derived hDPSC cultures. The number of biological replicates was as follows: (A) *n* = 6; (B) *n* = 4; (C) representative flow cytometry plots from *n* = 4; (D) *n* = 4; (E) representative images from *n* = 8; (F) *n* = 8; (G) representative images from *n* = 6; (H) *n* = 6; (I) *n* = 4.

To investigate the functional role of integrin signalling, hDPSCs were treated with the αvβ3 integrin antagonist; cilengitide. Treatment with 3 to 1000 nM cilengitide did not induce cytotoxicity (Figure 2A) and did not affect cell proliferation at days 1, 3, or 7 (Figure 2B). Based on these findings, a minimum dose of 3 nM and a maximum dose of 1000 nM cilengitide were selected for subsequent functional studies. Under basal culture conditions, cilengitide treatment did not significantly alter apoptotic activity at either concentration on day 3 (Figure 2C, D). Similarly, scratch wound assays demonstrated no significant effect of cilengitide on cell migration at 24 or 48 hours (Figure 2E, F).

The effect of cilengitide on osteogenic differentiation was evaluated by Alizarin Red S staining after 14 days of induction. All tested concentrations of cilengitide attenuated mineral deposition compared with control conditions (Figure 2G). Quantitative analysis revealed a significant reduction in mineralisation at concentrations of 80, 500, and 1000 nM (Figure 2H). To further assess osteogenic commitment at the molecular level, osteogenic-related gene expression was analysed on day 7. Treatment with 1000 nM cilengitide significantly downregulated *alkaline phosphatase (ALP)* and *collagen type I alpha 1 chain (COL1A1)* expression, whereas 3 nM had no significant effect. *RUNX2* expression exhibited a decreasing trend at 1000 nM but did not reach statistical significance (Figure 2I).

### Effect of cilengitide on Jagged1-treated hDPSCs

**Fig. 3:**
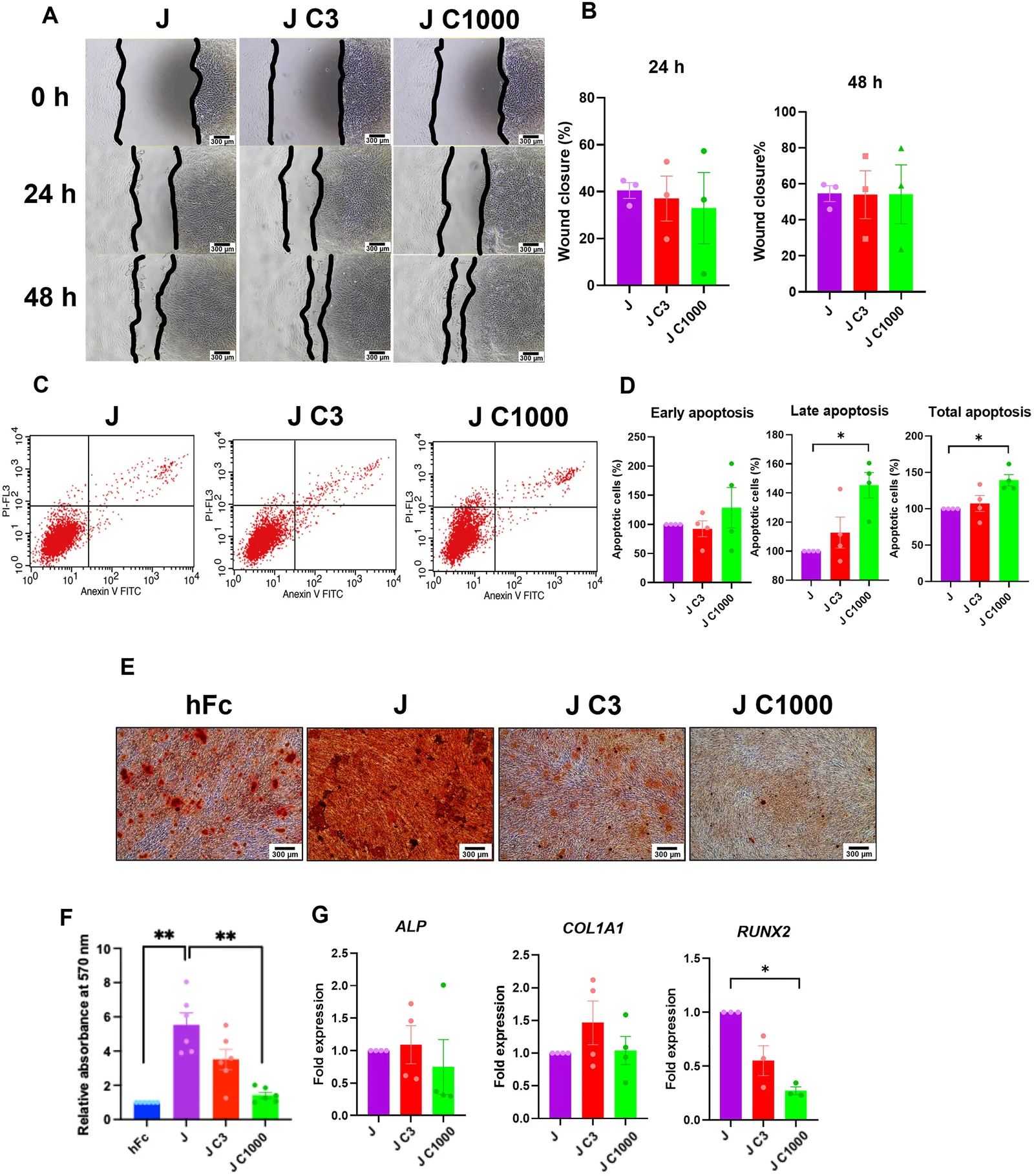
Effect of cilengitide on Jagged1-treated hDPSCs. (A and B) Cell migration of hDPSCs seeded on 10 nM Jagged1-coated surfaces following cilengitide treatment, as assessed by scratch wound assay at 24 and 48 hours. (C and D) Apoptotic activity of Jagged1-treated hDPSCs following cilengitide treatment on day 3, as determined by flow cytometry. (E) Alizarin Red S staining showing mineral deposition in Jagged1-treated hDPSCs under cilengitide treatment after 14 days of osteogenic induction. (F) Quantitative analysis of mineralisation in Jagged1-treated hDPSCs following cilengitide treatment. (G) qPCR analysis of *RUNX2* expression in Jagged1-treated hDPSCs under cilengitide treatment on day 7. Bars indicate significant differences between the treated groups and the control group (*P < .05, **P < .01). Biological replicates were independent donor-derived hDPSC cultures. The number of biological replicates was as follows: (A) representative images from *n* = 3; (B) *n* = 3; (C) representative flow cytometry plots from *n* = 4; (D) *n* = 4; (E) representative images from *n* = 6; (F) *n* = 6; (G) *n* = 4.

To investigate the requirement of integrin activity in Jagged1-induced responses, hDPSCs were seeded onto 10 nM indirect Jagged1-coated surfaces and treated with cilengitide. Cell migration analysis demonstrated that cilengitide treatment did not significantly affect the migratory capacity of hDPSCs on Jagged1-coated surfaces at either 24 or 48 hours (Figure 3A, B). Apoptosis analysis performed on day 3 revealed that treatment with 1000 nM cilengitide significantly increased late-stage apoptosis relative to the Jagged1-coated surface control, resulting in a significant increase in total apoptotic cells (Figure 3C, D).

The mineralisation potential of Jagged1-treated hDPSCs under integrin inhibition was assessed using Alizarin Red S staining and osteogenic gene-expression analysis. Cells seeded on Jagged1-coated surfaces and treated with 3 and 1000 nM cilengitide exhibited a dose-dependent decrease in mineralisation, with a significant reduction at 1000 nM compared with Jagged1 controls (Figure 3E, F). Consistent with these findings, qPCR analysis on day 7 revealed that *RUNX2* expression was significantly downregulated in hDPSCs treated with 1000 nM cilengitide on Jagged1-coated surfaces compared with Jagged1 controls (Figure 3G), indicating attenuation of Jagged1-induced osteogenic differentiation following functional integrin blockade. ERK1/2 phosphorylation was assessed at 15, 30, and 60 minutes following Jagged1 stimulation with or without cilengitide treatment. A variable increase in ERK1/2 phosphorylation was observed at selected time points following Jagged1 stimulation. However, cilengitide did not consistently alter this response (Figure S4A, B). Therefore, no definitive association between ERK1/2 phosphorylation and the inhibitory effect of cilengitide could be established.

### Effect of *ITGβ3* silencing on osteogenic differentiation and Jagged1-induced mineralisation

**Fig. 4:**
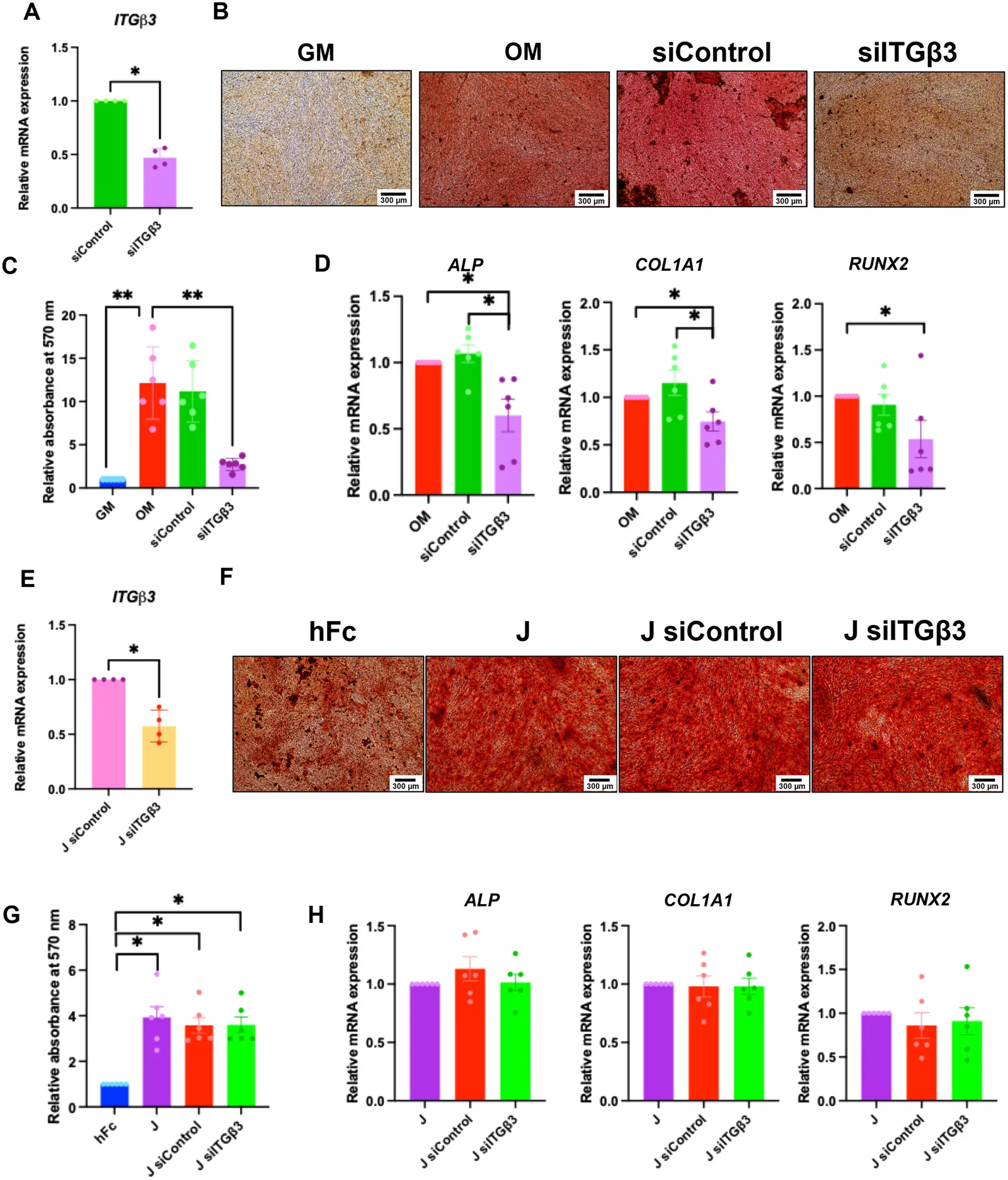
Effect of *ITGB3* silencing on osteogenic differentiation and Jagged1-induced mineralisation. (A) Confirmation of *ITGβ3* knockdown efficiency at the mRNA level in hDPSCs following siRNA transfection. (B and C) Alizarin Red S staining and quantitative analysis of mineral deposition in hDPSCs following *ITGB3* silencing under osteogenic induction conditions. (D) qPCR analysis of osteogenic-related gene expression on day 7 following *ITGB3* silencing. (E) Confirmation of *ITGB3* knockdown efficiency in hDPSCs cultured on Jagged1-coated surfaces following siRNA transfection. (F and G) Alizarin Red S staining and quantitative analysis of mineral deposition in Jagged1-treated hDPSCs following *ITGB3* silencing. (H) qPCR analysis of osteogenic-related gene expression in Jagged1-treated hDPSCs on day 7 following *ITGB3* silencing. Bars indicate significant differences between the treated groups and the control group (*P < .05, **P < .01). Biological replicates were independent donor-derived hDPSC cultures. The number of biological replicates was as follows: (A) *n* = 4; (B) representative images from *n* = 6; (C and D) *n* = 6; (E) *n* = 4; (F) representative images from *n* = 6; (G and H) *n* = 6.

To further investigate the specific role of *ITGβ3*, gene silencing was performed using *ITGB3*-targeting siRNA. Efficient knockdown of *ITGB3* expression was confirmed at the mRNA level on day 3 (Figure 4A). To confirm *ITGB3* knockdown at the protein level, immunofluorescence staining was performed following siITGB3 transfection. Representative images showed reduced ITGβ3 staining in si*ITGB3*-transfected hDPSCs compared with siControl-transfected cells (Figure S5A). Quantitative analysis of relative ITGβ3 fluorescence intensity, normalised to the siControl group, confirmed a significant reduction in ITGβ3 protein expression following si*ITGB3* transfection (Figure S5B). These findings support effective suppression of *ITGB3* expression at both the mRNA and protein levels. Under osteogenic induction conditions, silencing of *ITGB3* alone significantly reduced mineralisation after 14 days, as demonstrated by Alizarin Red S staining and quantitative analysis (Figure 4B, C). Consistently, gene expression analysis revealed that *alkaline phosphatase, COL1A1*, and *RUNX2* were significantly downregulated following *ITGB3* silencing (Figure 4D).

To verify the efficiency of *ITGB3* silencing under Jagged1 stimulation, *ITGB3* mRNA expression was examined in hDPSCs seeded onto indirectly immobilised Jagged1-coated surfaces following siRNA transfection. *ITGB3* expression remained significantly reduced in si*ITGB3*-transfected cells compared with siControl under Jagged1-coated conditions (Figure 4E), confirming effective knockdown on Jagged1-coated surface. Importantly, Jagged1 retained its ability to induce mineralisation in *ITGB3*-silenced hDPSCs, with no significant differences in calcium deposition (Figure 4F, G) or osteogenic gene expression (Figure 4H) observed compared with Jagged1-treated siControl cells. These findings indicate that *ITGB3* transcriptional silencing suppresses basal osteogenic differentiation but does not abrogate Jagged1-induced osteogenic differentiation.

## Discussion

Integrins are fundamental transmembrane receptors that regulate cell adhesion, mechanotransduction, and lineage commitment by linking ECM cues to intracellular signalling networks.^33^ Accumulating evidence demonstrates that integrin signalling influences MSC differentiation towards osteoblasts, chondrocytes, and adipocytes by integrating biochemical and biomechanical cues from the ECM.^34^ Among these receptors, the β3 integrin subunit, encoded by *ITGB3* and commonly incorporated into the αvβ3 heterodimer, has been implicated in osteogenesis, matrix mineralisation and bone remodelling, in part through ITGβ3–FAK–ERK–RUNX2 signalling in osteoprogenitors.^28^ Despite extensive investigation of bone marrow–derived MSCs and osteoblasts, the functional role of ITGβ3 in hDPSCs and in Notch-mediated osteogenic differentiation remains largely unexplored.

In the present study, Jagged1-mediated Notch activation significantly upregulated *ITGB3* mRNA expression and ITGβ3 protein levels in hDPSCs. This observation is consistent with previous reports demonstrating that Notch signalling regulates integrin expression in vascular smooth muscle cells and endothelial cells, in which Notch promotes integrin-dependent adhesion and maturation.^35^^,^^36^ In addition, Notch1 has been shown to activate β1 integrins via the small GTPase R-Ras, supporting a direct regulatory link between Notch signalling and integrin activation in mammalian cells.^37^ Consistent with this broader Notch–integrin relationship, the present RNA-seq reanalysis revealed enrichment of integrin-related and ECM organisation pathways among Jagged1-responsive genes. Moreover, activation of the canonical Notch target genes *HES1* and *HEY1* over the 7-day time course was positively associated with *ITGB3* expression, supporting a link between Notch target gene expression and ITGβ3 upregulation in Jagged1-treated hDPSCs. Collectively, these findings identify ITGβ3 as a candidate Notch-responsive integrin subunit in hDPSCs.

Jagged1 has previously been reported to activate canonical Notch signalling and promote odonto/osteogenic differentiation in multiple dental-derived stem cell populations.^11^^,^^14^^,^^16^^,^^17^ Therefore, the main contribution of this study is not to reconfirm the pro-osteogenic effect of Jagged1, but to extend previous findings by linking Jagged1-induced Notch activation to integrin-associated adhesion signalling. The present data suggest that Jagged1-induced osteogenic differentiation is accompanied by activation of an integrin-related molecular programme, with ITGβ3 emerging as one Notch-responsive component of this response.

A key finding of this study was the different outcomes observed following pharmacological integrin inhibition and *ITGB3* silencing. Cilengitide attenuated osteogenic differentiation under both basal and Jagged1-stimulated conditions, as demonstrated by reduced mineral deposition and osteogenic marker expression. In contrast, *ITGB3* silencing impaired basal osteogenic differentiation but did not significantly reduce Jagged1-induced mineralisation. This discrepancy indicates that the effects of cilengitide cannot be attributed solely to inhibition of ITGβ3.

Cilengitide is a cyclic RGD-containing antagonist with high affinity for αvβ3 and αvβ5 integrins.^38^ Its biological effects may therefore include disruption of multiple RGD-dependent cell–matrix interactions and associated adhesion functions rather than selective suppression of ITGβ3 alone.^23^^,^^39^ Previous studies have shown that cilengitide can impair integrin-mediated adhesion, suppress FAK/Src signalling, disrupt cytoskeletal organisation, and induce detachment-related cellular responses.^40^^,^^41^ The functional importance of αvβ3 in osteoblast proliferation, differentiation, and bone formation has also been demonstrated *in vivo*,^42^ underscoring the physiological significance of integrin-mediated adhesion beyond *ITGB3* transcription alone. In contrast, siRNA-mediated silencing reduces *ITGB3* expression without necessarily eliminating residual ITGβ3 protein activity or affecting other integrin heterodimers. Functional redundancy and crosstalk among integrins have been reported in other cellular contexts,^43^ and integrin expression can change dynamically during osteogenic differentiation, with several integrin subunits being regulated concurrently.^44^ However, the present study did not assess residual ITGβ3 activity or compensatory changes in other integrin subunits. Therefore, whether these mechanisms account for the preserved osteogenic response to Jagged1 following *ITGB3* knockdown remains to be determined in future studies using broader integrin profiling and targeted functional analyses.

ERK1/2 phosphorylation was evaluated as an exploratory downstream readout because integrin-associated ERK signalling has previously been linked to RUNX2 regulation and osteogenic differentiation.^29^ However, the ERK response following Jagged1 stimulation was variable, and cilengitide did not consistently suppress ERK1/2 phosphorylation. Therefore, the present findings do not support a definitive role for ERK in mediating the inhibitory effect of cilengitide on Jagged1-induced osteogenic differentiation. Further studies using pathway-specific inhibition and additional time points are required to determine whether ERK contributes to the Jagged1-associated osteogenic response.

It should also be noted that the reduction in mineralisation observed with high-dose cilengitide may be influenced by reduced cell survival, as increased apoptosis was observed under the 1000 nM cilengitide condition in the presence of Jagged1. Thus, the mineralisation data at this concentration may reflect combined effects on cell survival, adhesion-associated signalling, and osteogenic differentiation, rather than a differentiation-specific effect alone. Loss of integrin-mediated attachment is a well-recognised trigger of apoptosis in anchorage-dependent cells via caspase-dependent pathways.^45^ Therefore, the reduced mineral deposition at this concentration may partly result from a decrease in the number of surviving matrix-producing cells. However, cell detachment and apoptosis-related signalling were not directly examined in the present study and should be investigated in future studies.

Phosphoinositide-3-kinase/protein kinase B (PI3K/Akt) signalling is another candidate pathway for future investigation because it regulates stem cell survival, proliferation, and osteogenic commitment and can function downstream of integrin activation in mesenchymal and dental-derived stem cells.^46^, ^47^, ^48^ Both integrins and Notch receptors can independently influence PI3K/Akt activity, and crosstalk between these pathways has been reported in skeletal progenitor systems.^49^^,^^50^ However, PI3K/Akt signalling was not examined in the present study, and no conclusion can be drawn regarding its contribution to the observed effects. Future studies using pathway-specific inhibition and rescue experiments will be required to determine whether PI3K/Akt participates in the response to Jagged1 stimulation or cilengitide treatment.

From a translational perspective, hDPSCs are a readily accessible stem cell source with odontogenic and osteogenic potential.^51^^,^^52^ The present findings are limited to an *in vitro* mechanistic model and do not directly demonstrate the efficacy of a biomaterial-based strategy. Nevertheless, because RGD-containing motifs can support integrin-mediated cell attachment and osteogenic differentiation,^53^^,^^54^ future studies could investigate whether combining adhesion-supportive motifs with immobilised Jagged1 enhances hDPSC attachment, differentiation, and matrix mineralisation within regenerative scaffolds.

Several limitations of this study should be acknowledged. First, although hDPSCs were isolated from independent healthy adult donors and used as biological replicates, the study was not designed or powered to perform formal donor-stratified analyses according to sex, age, or baseline osteogenic potential. Therefore, donor-specific variation in Jagged1–integrin responses should be further investigated using larger donor cohorts. Second, all experiments were conducted *in vitro.* The clinical microenvironment of tissue repair involves bacterial biofilms, inflammatory mediators, immune cell responses, vascular changes, ECM remodelling and interactions among multiple resident cell populations, which are not fully represented in the present culture system. In addition, the treatment periods used in this study were selected to capture molecular, cellular and mineralisation endpoints under controlled culture conditions, rather than to directly mimic the clinical healing timeline. Thus, *in vivo* validation in dental pulp regeneration or alveolar bone healing models will be essential to confirm the therapeutic relevance of integrin–Notch coupling. Third, cilengitide was used as a pharmacological inhibitor of αvβ3-associated adhesion; however, pharmacological inhibition may affect multiple RGD-binding integrin interactions and cannot be interpreted as evidence of *ITGβ3*-specific dependency. Fourth, although *ITGB3* knockdown was confirmed at both the mRNA and protein levels, changes in other integrin subunits were not assessed. ERK phosphorylation was examined only as an exploratory downstream readout, and cilengitide did not consistently suppress the observed ERK response. Other adhesion-related pathways, including FAK and Akt, were not evaluated. These limitations preclude identification of the specific signalling components responsible for the observed effects. Future studies should address these questions through broader integrin profiling, targeted pathway inhibition, and rescue experiments.

In conclusion, Jagged1-mediated Notch activation increased ITGβ3 expression in hDPSCs, whereas cilengitide attenuated both basal and Jagged1-induced osteogenic differentiation. In contrast, *ITGB3* silencing reduced basal osteogenesis but did not significantly impair the osteogenic response to Jagged1. These contrasting findings indicate that the effect of cilengitide is unlikely to result from ITGβ3 inhibition alone. Interpretation of the high-dose cilengitide findings should also consider the accompanying increase in apoptosis. Further studies examining additional integrin subunits and downstream adhesion pathways are required to define the molecular basis of the Jagged1-associated osteogenic response.

## Author contributions

*Contributed to data acquisition, data analysis and interpretation, drafted the manuscript, and critically revised the manuscript:* Kornsuthisopon. *Contributed to data interpretation and critically revised the manuscript:* Chansaenroj. *Contributed to data acquisition:* Phothichailert. *Critically revised the manuscript:* Gedik, Zhang, Yuan, Silvio. *Contributed to data acquisition and critically revised the manuscript*: Nowwarote. *Contributed to the conception and design of the study, data interpretation, and critical revision of the manuscript*: Osathanon. *Final approval of the version to be published and agree to be accountable for all aspects of the work:* All authors.

## Ethics statement

The study was approved by the Human Research Ethics Committee of the Faculty of Dentistry, Chulalongkorn University, Bangkok, Thailand (approval no. 02/2025, date of approval: 10 January, 2025).

## Declaration of generative AI and AI-assisted technologies in the writing process

During the revision phase of this manuscript, the authors utilised generative AI tools to enhance the clarity and quality of the English language in select paragraphs. All revisions made using the tool were subsequently reviewed and edited by the authors to ensure accuracy and integrity. The authors assume full responsibility for the final content of the manuscript.

## Funding

This project was supported by a Faculty Research Grant (DRF 68_004), the Faculty of Dentistry (to T.O.) and The Ratchadaphiseksomphot Endowment Fund, Chulalongkorn University (The Exchange Faculty Travel Grant; Grant No. CTG168031, awarded to C.K.). The funders had no role in the study design, data collection, analysis, publication decision, or manuscript preparation.

## Conflict of interest

Thanaphum Osathanon and Chengfei Zhang are Associate Editors of this journal and were not involved in the editorial review or the decision to publish this article. Other authors declare that they have no known competing financial interests or personal relationships that could have appeared to influence the work reported in this article.

## Appendix Supplementary materials

Image, application 1

## Footnotes

Supplementary material associated with this article can be found in the online version at doi:10.1016/j.identj.2026.109808.
