# Supplementary material for: Integrin-Associated Signalling Regulates Jagged1-Induced Osteogenic Differentiation of Human Dental Pulp Stem Cells
Source: Int Dent J. 2026 Aug 17;76(5):109808. doi: 10.1016/j.identj.2026.109808 (PMC13508636; doi:10.1016/j.identj.2026.109808)
Supplement: Supplementary file 1 [file mmc1.docx]

**Supplementary Data File**

**Integrin-Associated Signalling Regulates Jagged1-Induced Osteogenic Differentiation of Human Dental Pulp Stem Cells**

**GM**

**300 µm**

**OM**

**300 µm**

**GM**

**30 µm**

**ADM**

**30 µm**

**A**

**C**

**B**

**Figure S1.** Characterisation of hDPSCs isolated from dental pulp tissues. (A) Expression of MSC surface markers (CD44, CD105, and CD90) and the hematopoietic stem cell marker (CD45) was evaluated by flow cytometry. (B) Osteogenic differentiation of hDPSCs after 14 days of induction, as demonstrated by Alizarin Red S staining of mineralised nodules (scale bar = 300 μm). (C) Adipogenic differentiation of hDPSCs after 16 days of induction, showing intracellular lipid droplet accumulation detected by Oil Red O staining (scale bar = 30 μm). Biological replicates were independent donor-derived hDPSC cultures (n = 3).

**A**

**B**

**Figure S2.** Correlation between Notch target gene expression and ITGβ3 expression following Jagged1 stimulation. Exploratory Spearman correlation analysis was performed using Jagged1-treated hDPSC samples collected on days 1, 3 and 7. (A) Correlation between *HES1* and *ITGβ3* expression. (B) Correlation between *HEY1* and *ITGβ3* expression. Biological replicates were independent donor-derived hDPSC cultures (n = 8), with samples collected at three time points. Each dot represents one donor-derived culture at one time point.

**B**

**A**

**Figure S3.** Expression of endogenous Notch pathway components following immobilised Jagged1 stimulation. hDPSCs were cultured on hFc- or 10 nM Jagged1-coated surfaces for 24 h, with or without DAPT pretreatment. qPCR analysis was performed to evaluate endogenous expression of (A) *JAG1* and (B) *NOTCH1.* Expression levels were normalised to the hFc control group. Biological replicates were independent donor-derived hDPSC cultures (n = 4).

**Figure S4.** ERK1/2 phosphorylation following Jagged1 stimulation and cilengitide treatment. hDPSCs were cultured on Jagged1-coated surfaces with or without cilengitide treatment for 15, 30, and 60 minutes. (A) Representative western blot images of phosphorylated ERK1/2, total ERK1/2, and β-actin in hDPSCs cultured on Jagged1-coated surfaces with or without cilengitide at designated timepoints. (B) Quantification of phosphorylated ERK1/2 relative to total ERK1/2.Bars indicate a significant difference between the treated groups and the control group (* *p* < 0.05). Biological replicates were independent donor-derived hDPSC cultures (n = 3).

**A**

**B**

**Figure S5.** Protein-level validation of ITGβ3 knockdown. hDPSCs were transfected with control siRNA (siControl) or ITGβ3-targeting siRNA (siITGβ3), followed by immunofluorescence staining for ITGβ3. (A) Representative immunofluorescence images showing ITGβ3 expression in siControl- and siITGβ3-transfected hDPSCs. ITGβ3 is shown in red and nuclei are counterstained with DAPI in blue. Scale bar = 20 µm. (B) Quantification of relative ITGβ3 fluorescence intensity normalized to the siControl group. Bars indicate a significant difference between the treated groups and the control group (* *p* < 0.05). Biological replicates were independent donor-derived hDPSC cultures (n = 4).

**Supplementary Table 1** The oligonucleotide sequences

| **Gene** | **Forward** | **Reverse** | **Accession number** |
| --- | --- | --- | --- |
| *GAPDH* | 5’-TCATGGGTGTGAACCATGAGAA-3’ | 5’-GGCATGGACTGTGGTCATGAG-3’ | NM_001357943.2 |
| *ALP* | 5’-CGAGATACAAGCACTCCCACTTC-3’ | 5’-CTGTTCAGCTCGTACTGCATGTC-3’ | NM_000478.3 |
| *COL1A1* | 5′-GTGCTAAAGGTGCCAATGGT-3′ | 5′-ACCAGGTTCACCGCTGTTAC-3′ | NM_000088.3 |
| *HES1* | 5’-AGGCGGACATTCTGGAAATG-3’ | 5’-CGGTACTTCCCCAGCACACTT-3’ | NM_005524.2 |
| *HEY1* | 5’-CTGCAGATGACCGTGGATCA-3’ | 5’-CCAAACTCCGATAGTCCATAGCA-3’ | NM_012258.3 |
| *ITGα1* | 5’-TGGTGGTGCTGCCCTCTTCTG-3’ | 5’-GTGAATCTAGGGTGACACGGTACTGC-3’ | NM_181501.2 |
| *ITGα5* | 5’-TGCCTCCCTCACCATCTTC-3’ | 5’-TGCTTCTGCCAGTCCAGC-3’ | NM_002205.5 |
| *ITGα6* | 5’-GCTGGTTATAATCCTTCAATATCAATTGT-3’ | 5’-TTGGGCTCAGAACCTTGGTTT-3’ | NM_001079818.3 |
| *ITGβ3* | 5’-TTGGAGACACGGTGAGCTTC-3’ | 5’-TTAGGTTCAGCTTGGGCCTG-3’ | NM_000212.3 |
| *RUNX2* | 5’-ATGATGACACTGCCACCTCTGA-3’ | 5’-GGCTGGATAGTGCATTCGTG-3’ | NM_001024630.3 |
